# Curcumin suppresses ovalbumin-induced allergic conjunctivitis

**Published:** 2012-07-18

**Authors:** So-Hyang Chung, Seong Hyun Choi, Jin A. Choi, Roy S. Chuck, Choun-Ki Joo

**Affiliations:** 1 Department of Ophthalmology and Visual Science, College of Medicine, The Catholic University of Korea, Seoul, Korea; 2 Department of Ophthalmology and Visual Sciences, Albert Einstein College of Medicine and Montefiore Medical Center, Bronx, NY; 3 Catholic Institute of Visual Science, College of Medicine, The Catholic University of Korea, Seoul, Korea

## Abstract

**Purpose:**

Allergic conjunctivitis (AC) from an allergen-driven T helper 2 (Th2) response is characterized by conjunctival eosinophilic infiltration. Because curcumin has shown anti-allergic activity in an asthma and contact dermatitis laboratory models, we examined whether administration of curcumin could affect the severity of AC and modify the immune response to ovalbumin (OVA) allergen in an experimental AC model.

**Methods:**

Mice were challenged with two doses of topical OVA via the conjunctival sac after systemic sensitization with OVA in aluminum hydroxide (ALUM). Curcumin was administered 1 h before OVA challenge. Several indicators for allergy such as serum immunoglubulin E (IgE) antibodies production, eosinophil infiltration into the conjunctiva and Th2 cytokine production were evaluated in mice with or without curcumin treatment.

**Results:**

Mice challenged with OVA via the conjunctival sac following systemic sensitization with OVA in ALUM had severe AC. Curcumin administration markedly suppressed IgE-mediated and eosinophil-dependent conjunctival inflammation. In addition, mice administered curcumin had less interleukin-4 (IL-4) and interleukin-5 (IL-5) (Th2 type cytokine) production in conjunctiva, spleen, and cervical lymph nodes than mice in the non-curcumin-administered group. OVA challenge resulted in activation of the production of inducible nitric oxide (iNOS), and curcumin treatment inhibited iNOS production in the conjunctiva.

**Conclusions:**

We believe our findings are the first to demonstrate that curcumin treatment suppresses allergic conjunctival inflammation in an experimental AC model.

## Introduction

Allergic conjunctivitis (AC) describes a group of conditions ranging from mild to severe [1]. The immunopathogenic mechanisms in these allergic disorders involve a combination of immunoglobulin E (IgE)-mediated and T helper 2 (Th2) cell-mediated responses [2-4]. The IgE-mediated conjunctival allergic reaction can be reproduced easily by specific conjunctival provocation [5], which results an early reaction followed by a predominant infiltration of eosinophilic inflammatory cells [6,7]. Eosinophils are the hallmark of allergic disease, particularly in severe chronic ocular allergy where they are easily found in quantity in tears and tissues. The release of eosinophil granule proteins is implicated in the pathogenesis of conjunctival inflammation.

Curcumin, which imparts the yellow color to curry, is a natural product of the spice turmeric, *Curcuma longa* L (Zingiberaceae). Curcumin exhibits a variety of pharmacologic activities, including anti-inflammatory, anti-cancer and anti-oxidant effects [8-11]. Curcumin also possesses anti-allergic activity in animal models of allergy [12-15]. Several researchers have shown that curcumin inhibits inducible nitric oxide (iNOS) [8,9,15] and inflammatory cytokines induced by macrophages and dendritic cells [10,16].

In this study, we evaluated the anti-allergic activity of curcumin in an experimental AC model. The administration of curcumin markedly suppressed IgE production, eosinophil-dependent conjunctival inflammation and inhibited Th2 type immune responses. Our findings for the first time demonstrate that curcumin attenuates Th2 cell-mediated allergic conjunctivitis in an experimental AC model.

## Methods

### Protocol for mouse model of experimental allergic conjunctivitis (EAC)

**Figure 1:**
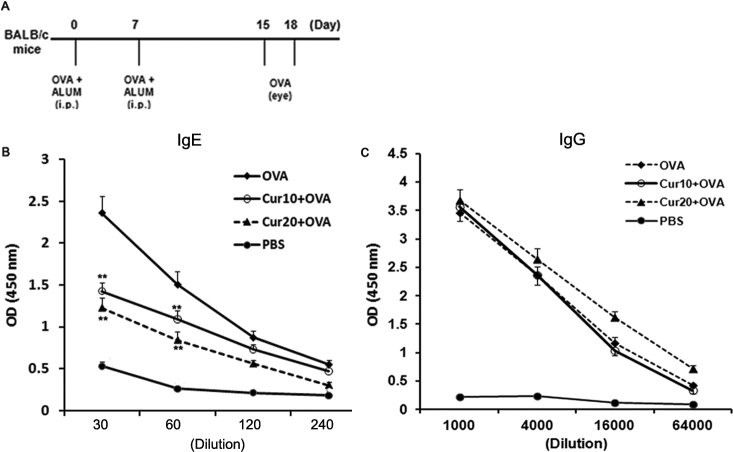
Selective reduction of IgE antibody secretion in serum of curcumin (Cur)-treated mice. **A**: Experimental protocol. BALB/c mice were injected intraperitoneally (i.p.) with 1 μg of ovalbumin (OVA) and 200 μl of 1.5% ALUM on days 0 and 7. Curcumin was administered i.p. One h before OVA challenge into conjunctival sac on days 15 and 18. **B**: Administration of curcumin before OVA challenge led to a profound decrease of Ag-specific IgE antibody secretion in serum. **C**: Administration of curcumin before OVA challenge did not decrease of Ag-specific IgG antibody secretion in serum. **, p<0.01 (n=6 in each group, three independent experiments).

Our study was approved by the Catholic University of Korea Institutional Animal Care and Use Committee. Wild-type (WT) BALB/c mice (4- to 5-wk-old females) were purchased from Charles River Laboratories (Orient Co., Sungnam, Korea). To generate EAC, mice were sensitized intraperitoneally (i.p.) with 1 μg of ovalbumin (OVA; Grade V; Sigma-Aldrich, St. Louis, MO) and 200 μl of 1.5% aluminum hydroxide (ALUM; Pierce, Rockford, IL) on days 0 and 7, and then challenged two times topically in the conjunctival sac with 250 μg of OVA on days 15 and 18 (Figure 1A) [17,18]. Control mice were given OVA with ALUM in sensitization stages and PBS in place of OVA in challenge stages. Twenty-four h after the final challenge with OVA, mice were given a fatal dose of ketamine and blood, eyes, spleen, and cervical lymph node (CLN) were collected.

### Administration of curcumin

Curcumin solution (10 or 20 mg/kg bodyweight/day, Sigma-Aldrich) was freshly prepared and administered i.p. twice on days 14 and 17, beginning 1 h before the challenge in the conjunctival sac.

### Evaluation of eosinophilic infiltration

The eyes including eyelids and conjunctivae were exenterated. After harvest they were fixed in 10% buffered formalin, cut into horizontal 4-μm-thick sections, and stained with acid-giemsa for detection of eosinophils [17,18]. OVA-specific AC in this mouse model develops in an eosinophil-dependent manner not in mast cells [17,19]. In each section, infiltrating cells in the lamina propria mucosae of the tarsal and bulbar conjunctivas were counted by two masked observers [17,18]. The sections counted were those of the central portion of the eye, which included the pupil and optic nerve head. The data are presented as mean±standard deviation (SD) per slide.

### Enzyme-linked immunosorbent assay (ELISA)* f*or OVA-specific IgE and IgG antibodies in serum

Twenty-four h after OVA challenge of immunized mice, blood was collected and serum prepared. Briefly, the immunoplates (Nalge Nunc International, Naperville, IL) were coated with OVA (1 mg/ml) overnight at 4 °C. After blocking with 1% BSA in PBS for 1h at room temperature, serial dilutions of serum samples were added and incubated for 4 h at room temperature. The plates were then washed with PBS plus 0.05% Tween (PBS/T) and incubated for 2 h at room temperature with horseradish peroxidase (HRP)-conjugated rat anti-mouse IgE or IgG antibodies (Southern Biotech, Birmingham, AL). After a wash with PBS/T, color reaction was developed with 3, 3‘, 5, 5‘-tetramethyl-benzidine (Moss Inc., Pasadena, CA) and stopped with 0.1N HCl [17,19].

### Lymphoid cell culture and cytokine analysis

Spleens and cervical lymph nodes (CLN) were harvested, and a single cell suspension was cultured in RPMI 1640 medium supplemented with 2 mM L-glutamine, 50 mM 2-mercaptopurine, and 10% heat-inactivated fetal calf serum (all from Invitrogen Life Technologies, Carlsbad, CA). Cells were cultured at 5×10^6^ cells/ml (spleen) or 1×10^6^ cells/ml (CLN) with 1 mg/ml OVA for 96 h in 96-well plates (Nunc, Rochester, NY).

Th2 cytokine levels (including IL-4 and IL-5) of culture supernatants were assayed using a Cytometric Bead Array Mouse Th1/Th2 Cytokine kit (BD PharMingen, San Diego, CA).

### Immunohistochemical analysis

The eyes including eyelids and conjunctivae, spleens, and CLN were fixed in 10% buffered formalin, cut into horizontal 4-μm-thick sections. Four-μm sections were dewaxed by immersion in xylene (twice for 5 min each time) and hydrated by serial immersion in 100%, 90%, 80%, and 70% ethanol and PBS. Antigen retrieval was performed by microwaving sections for 20 min in Target Retrieval Solution (DAKO, Carpinteria, CA). Sections were washed with PBS (twice for 10 min each time), and blocking buffer (10% BSA in PBS) was added for 1 h. Sections were incubated with primary antibodies in blocking buffer overnight at 4 °C. The primary antibodies used for this study included rat anti-mouse IL-4 (clone 11B11) from BD PharMingen and rat anti-mouse IL-5 (clone TRFK5) from BioLegend (San Diego, CA). After extensive washing, the sections were incubated with Alexa Fluor 488–labeled goat anti-mouse antibody (1:200; Molecular Probes, Eugene, OR) for 1 h. The sections were washed with 0.01 M PBS three times and mounted in Vectashield mounting medium with DAPI (Vector Laboratories, Burlingame, CA). The sections were viewed with a fluorescence microscopy (Axio Imager; Carl Zeiss Meditec, Jena, Germany).

### Western blot analysis

Total lysates from conjunctiva were loaded onto SDS PAGE, transferred to Immobilion^TM^-P (Millipore Corp., Bedford, MA) PVDF membranes, and blotted with anti- inducible nitric oxide (iNOS) antibody (BD Biosciences). Antibody was incubated with substrate overnight at 4 °C and blotted with specific HRP-conjugated secondary antibodies purchased from Amersham Pharmacia Biotech (Buckinghamshire, England). We used Quantity One 1-D Analysis Software (Bio-Rad, Hercules, CA) to quantify the amount from each sample.

### Statistical analysis

Statistical significance between groups was examined by one-way ANOVA followed by Tukey test using SPSS version 12.0 (SPSS Inc., Chicago, IL). Data are representative of three independent experiments with six mice in each group. In this study a p<0.05 was considered significant.

## Results

### Curcumin treatment selectively inhibits serum IgE antibody production in EAC

To investigate the notion that curcumin treatment modulates adaptive immune responses to conjunctival allergens, BALB/c mice were sensitized i.p. with OVA and ALUM at days 0 and 7. They were then injected i.p. with curcumin solution (10 or 20 mg/kg bodyweight/day) before two challenges via the conjunctival sac with OVA as shown in Figure 1A. To clarify the severity of EAC in mice administered curcumin solution, we assessed levels of OVA-specific IgE antibodies in sera. A combination of systemic priming and local boosting with OVA resulted in significantly higher levels of Ag-specific IgE antibody secretion (Figure 1B). However, the administration of curcumin before OVA challenge into the conjunctival sac led to a profound decrease of Ag-specific IgE antibody secretion (Figure 1B). To address overall immunosuppression by curcumin, we also assessed levels of OVA-specific IgG antibodies in sera. OVA-specific IgG antibody levels in sera did not decrease after administration of curcumin in EAC model (Figure 1C). Taken together, analyses of IgE levels in mice administered curcumin suggest that curcumin suppressed allergen specific IgE production not overall immune responses.

### Curcumin treatment attenuates OVA-induced allergic conjunctival inflammation in EAC

**Figure 2:**
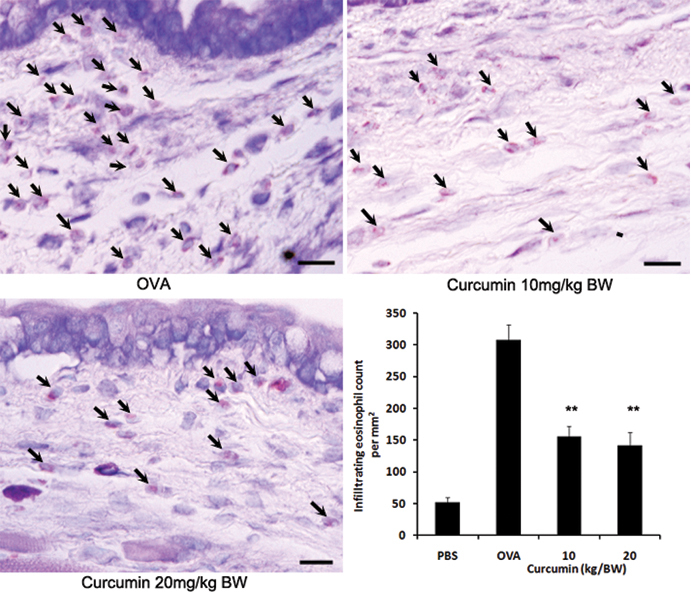
Curcumin administration prevents development of allergic conjunctival inflammation. Infiltration of eosinophils into the conjunctiva declined in curcumin-treated mice. Bars=10 μm. **, p<0.01 (n=6 in each group, three independent experiments). BW, bodyweight.

In terms of conjunctival inflammation, histologic findings demonstrated significant infiltration of eosinophils in the conjunctiva of OVA-challenged mice after systemic priming (Figure 2). Interestingly, eosinophils in conjunctiva after allergen challenge were decreased in mice administered curcumin. Eosinophil counts were not significantly different between mice injected with differing curcumin regimens (10 or 20 mg/kg bodyweight/day; Figure 2). These results indicate that curcumin suppressed eosinophilic infiltration in OVA-induced allergic model.

### Curcumin treatment inhibits Th-2 type cytokine responses in EAC

**Figure 3:**
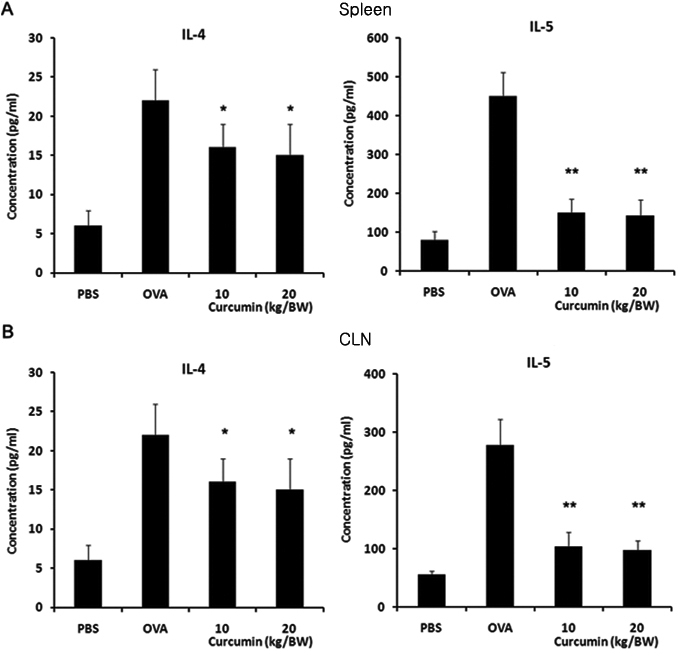
Administration of curcumin before ovalbumin (OVA) challenge reduces Th2-type immune responses in an OVA-induced allergic conjunctivitis model. Splenocytes and monocytes from cervical lymph nodes (CLN) were harvested on day 19 and cultured with 1 mg/mL OVA in vitro. Culture supernatants were collected at 96 h and cytokine concentration was determined by FACS analysis. Splenocytes (**A**) and monocytes from CLN (**B**) from curcumin-treated mice produced significantly less IL-4 and IL-5 (Th2-type cytokine) compared with those from untreated mice. * p<0.05, ** p<0.01 (n=6 in each group, three independent experiments). BW, bodyweight.

**Figure 4:**
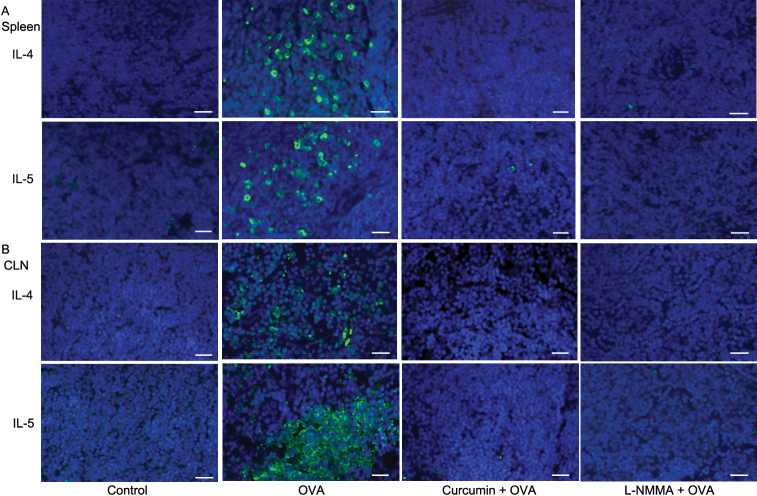
Curcumin administration (20 mg/kg bodyweight/day) decreases infiltration of IL-4 and IL-5 producing cells in the spleens and cervical lymph nodes (CLN) of mice in an ovalbumin (OVA)-induced allergic conjunctivitis model. The iNOS inhibitor, L-NMMA, administration also decreases infiltration of cells. Representative images of immunohistochemical staining for IL-4 and IL-5 in the spleens (**A**) and CLN (**B**) were demonstrated. Bar=20 μm.

**Figure 5:**
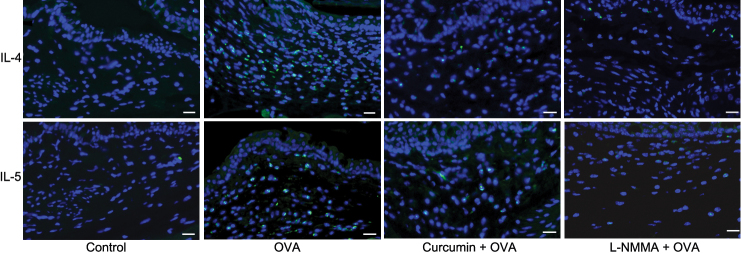
Curcumin administration (20 mg/kg bodyweight/day) decreases infiltration of IL-4 and IL-5 producing cells in the conjunctiva of mice in an ovalbumin (OVA)-induced allergic conjunctivitis model. The iNOS inhibitor, L-NMMA, administration also decreases infiltration of cells. Representative images of immunohistochemical staining for IL-4 and IL-5 in conjunctiva stroma were demonstrated. Bar=20 μm.

To determine the role of curcumin before OVA challenge in Th2 cells, we analyzed Th2-type cytokine secretions by mononuclear cells. Splenocytes isolated from mice with AC elicited significantly higher levels of Th2-type cytokines (i.e., IL-4 and IL-5) after re-stimulation with OVA in vitro than those from the control group. As expected, splenocytes and mononuclear cells in CLN isolated from curcumin-injected mice produced significantly less IL-4 and IL-5 compared with those from mice challenged with OVA alone (Figure 3). Immunostaining data confirmed that IL-4 and IL-5 producing cells infiltrated into the spleens and CLN in mice challenged with OVA alone and curcumin suppressed infiltration of these cells (Figure 4). Immunostaining data of the conjunctival stroma also demonstrated that curcumin suppressed infiltration of IL-4 and IL-5 producing cells in mice challenged with OVA (Figure 5). We observed that the administration of iNOS inhibitor, NG-Monomethyl-L-arginine, monoacetate salt (L-NMMA), also inhibited the infiltration of IL-4 and IL-5 producing cells. Taken together, these data demonstrate that the administration of curcumin before OVA challenge is committed to the inhibition of Th-2 type responses.

### Curcumin treatment inhibits iNOS production in EAC

**Figure 6:**
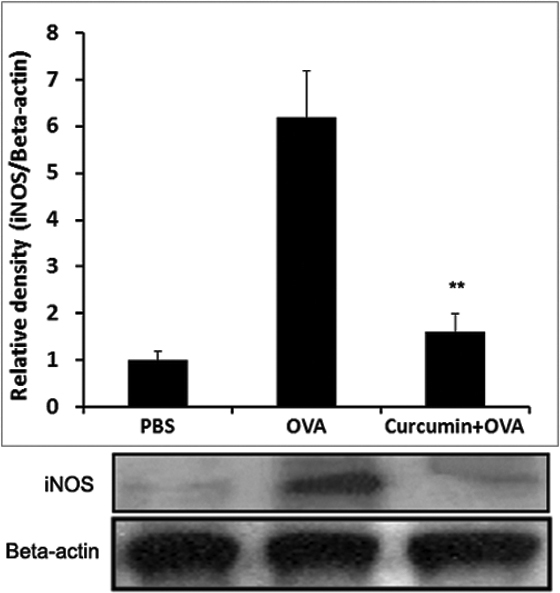
Curcumin administration (20 mg/kg bodyweight/day) decreases iNOS expression in the conjunctiva of mice in an ovalbumin (OVA)-induced allergic conjunctivitis model. Total lysates were prepared from the conjunctiva, and proteins were subjected to SDS–PAGE followed by western blot analysis with specific antibodies against iNOS. ** p<0.01 (n=6 in each group, three independent experiments).

To investigate further whether the levels of nitric oxide (NO) are related to curcumin-induced anti-allergic effects, we investigated iNOS expression using western blot analysis in conjunctiva. The same amount of proteins was used to compare groups. As shown Figure 6, the increased iNOS expression was observed in conjunctiva of OVA-challenged mice after systemic priming. However, the increased expression was significantly inhibited by curcumin (20 mg/kg bodyweight/day) treatment. The result indicates that curcumin-induced anti-allergic effects are related to the down-regulation of iNOS in EAC.

## Discussion

Curcumin is a yellow pigment isolated from *Curcuma longa* L (turmeric), a powerful anti-inflammatory agent [8-15]. However, to our knowledge, the effect of curcumin on allergic conjunctival inflammation has not been previously investigated. Our findings provide strong evidence for the anti-allergic effect of curcumin treatment in an OVA-induced EAC model and show that curcumin significantly reduces Th2-driven allergic conjunctival inflammation. Curcumin reduced Th2-type immune responses as reflected in the cytokine profile and Th2-type cell infiltration, production of antigen-specific IgE antibody, and recruitment of eosinophils characterized in an OVA-induced EAC model. We first demonstrated that curcumin decreases the expression of iNOS in this model.

The main pathophysiological changes in conjunctival allergic reactions include increased levels of IgE antibody in serum and infiltration of eosinophils into the conjunctiva [1]. These pathologic processes of allergic reaction are thought to be mediated by Th2-type cells, which preferentially produce IgE-enhancing cytokines such as IL-4 and IL-5 [2,3,20]. Indeed, our results provide direct evidence that cytokine synthesis by splenocytes, mononuclear cells from CLN and conjunctiva are predominantly of the Th2 type (IL-4 and IL-5). Such Th2-type cytokine synthesis contributes to the high levels of IgE antibody production and infiltration of eosinophils into the conjunctiva of OVA-challenged mice.

In this study, sensitization and challenge of BALB/C mice with OVA resulted in IgE-specific antibody response, Th2 cytokine production, and eosinophilic infiltrations into the conjunctiva. Our group previously demonstrated that the development of an allergic inflammatory reaction in the late phase of AC is mediated by eosinophils but not by mast cells in an OVA-induced EAC model [17]. Of interest, mice administered curcumin immediately before allergen challenge showed decreased serum OVA-specific IgE antibodies, eosinophil infiltration in conjunctiva, and IL-4 and IL-5 production in the OVA-induced EAC model. The administration of curcumin inhibited Th2-type cell differentiation, which led to decrease infiltration of IL-4 and IL-5 producing cells in spleen, CLN, and conjunctival stroma.

NO is a small-membrane permeable gas that serves as a mediator of many physiologic events. iNOS produces high amounts of NO, which implies cytotoxic and immune reactions [21]. Excessive NO may recruit eosinophils in the airway and shift the balance toward Th2 cells, thus exacerbating airway inflammation [15]. NO level and clinical allergic symptoms were found to be related in a rabbit experimental AC model [22,23] and NO inhibitors block eosinophil recruitment in the lungs [24,25]. In OVA-induced asthma model, potent iNOS inhibitor, curcumin treatment resulted in decreased production of iNOS in lung tissue, inflammatory cytokines, recruitment of eosinophils to lung airways [15]. Our study in an OVA-induced EAC model clearly demonstrated that OVA challenge results in activation of the production of iNOS in conjunctiva, increase of eosinophil infiltration into the conjunctiva, and enhance Th2 immune responses. The administration of curcumin before OVA challenge into the eye impaired such Th2-mediated allergic responses as eosinophilic infiltration into conjunctiva, Th2-type cytokine secretion, and serum levels of OVA-specific IgE antibodies. The iNOS inhibitor, L-NMMA, administration also inhibited infiltration of Th2-type cells in spleen, CLN, and conjunctival stroma in our EAC model.

This study showed the effect of systemic administered curcumin in allergic conjunctivis model. To evaluate local effect of curcumin, local use of curcumin via conjunctival sac or subconjunctival injection would be performed in the near future.

In summary, we found that curcumin attenuates allergic conjunctival inflammation in an OVA-induced EAC model. Therefore, it seems reasonable to consider that curcumin may prove useful as an adjunctive therapy for humans with AC.
